# Repeatable change in electrical resistance of Si surface by mechanical and electrical nanoprocessing

**DOI:** 10.1186/1556-276X-9-455

**Published:** 2014-08-31

**Authors:** Shojiro Miyake, Shota Suzuki

**Affiliations:** 1 Department of Innovative System Engineering, Nippon Institute of Technology, 4-1 Gakuendai, Miyashiro-machi, Saitama 345-8501, Japan

**Keywords:** Electrical resistance, Atomic force microscopy, Nanoprocessing, Mechanical processing

## Abstract

The properties of mechanically and electrically processed silicon surfaces were evaluated by atomic force microscopy (AFM). Silicon specimens were processed using an electrically conductive diamond tip with and without vibration. After the electrical processing, protuberances were generated and the electric current through the silicon surface decreased because of local anodic oxidation. Grooves were formed by mechanical processing without vibration, and the electric current increased. In contrast, mechanical processing with vibration caused the surface to protuberate and the electrical resistance increased similar to that observed for electrical processing. With sequential processing, the local oxide layer formed by electrical processing can be removed by mechanical processing using the same tip without vibration. Although the electrical resistance is decreased by the mechanical processing without vibration, additional electrical processing on the mechanically processed area further increases the electrical resistance of the surface.

## Background

In the future, a wide variety of nanoelectronic devices and nanomachines will be manufactured using nanofabrication techniques. Scanning probe microscopy (SPM) techniques are expected to be effective for the nanofabrication of nanometer-scale materials and devices [1] via the atomic-scale processing of nanostructures. Various attempts have also been made to use SPM techniques for the local modification of surfaces [2-5]. The so-called local oxidation technique is being investigated for the fabrication of devices on the nanometer scale [6-8]. In this method, the oxidizing species contained in the water layer adsorbed on the surface drift across the oxide layer under the influence of a high electric field produced by a voltage applied to the probe. This SPM-generated oxide layer can function as a mask for etching or can be used directly as an insulating barrier.

Nanometer-scale protuberances and grooves can be produced on a silicon surface by diamond tip sliding in the atmosphere using an atomic force microscopy (AFM). In some cases, when a diamond tip is slid on Si, the surface protuberates. This upheaval phenomenon has been applied to processing, and the nanoprotuberance characteristics and formation mechanism were evaluated [9,10]. Areas on a silicon (100) surface nanoprocessed by mechanical local oxidation through diamond tip sliding without a bias voltage have been found to act as an etching mask towards aqueous potassium hydroxide (KOH) solution [11-13]. Mechanical processing-induced mask patterns formed of such plastically deformed damaged layers are able to withstand selective wet etching processes for pattern transfer, resulting in the so-called maskless patterning [14,15] or friction-induced fabrication [16-18], and their formation mechanisms have been evaluated.

In our previous work investigating the fabrication of silicon nanostructures by mechanical local oxidation, silicon (100) specimens were processed by diamond tip sliding at low and high scanning densities to control their subsequent rate of etching by KOH solution. Processing at a low scanning density resulted in the removal of the natural oxide layer by the mechanical action. The thick mechanochemically oxidized layer formed suppressed etching by the KOH solution and decreased the etching rate without plastic deformation [19,20]. These results showed that etching depth can be controlled using etching time via natural oxide layer removal and mechanochemical oxide layer formation. These oxide layer removal and formation processes can be exploited to realize low-damage mask patterns [21].

An understanding of the electrical properties of areas processed by various mechanical and electrical processing methods is important to investigate their mechanism and to improve nanoprocessing techniques. Conductive atomic force microscopy (CAFM) can be used to obtain the local conductivity distribution of a surface [22]. We previously evaluated the profiles and electrical properties of mechanically and electrically processed areas on silicon using an electrically conductive diamond tip with and without electric voltage. A dense oxide layer was obtained after complex processing involving both mechanical and electrical processing, and the resulting decrease in current was found to be more significant than that caused by mechanical or electrical oxidation alone [23]. If repeatable change in the electrical resistance of Si surfaces were to be performed by combining such mechanical and electrical processing techniques, the method would find applications in the future nanodevice fabrication processes and nanolithography technology. For example, reproducible repeatedly recordable AFM memory could be fabricated, and direct formation and correction of circuit patterns for various electronic devices could be performed.

In this study, we attempted to alter the electrical resistance of a Si surface by forming and removing its oxide layer using electrical and mechanical processing. The modifications were performed by scanning the Si surface with an electrically conductive diamond tip using the mechanical and electrical methods with and without vibration. The profile, electric current, and friction distributions of the mechanically and electrically processed areas were characterized. The effects of vibration on (changes in) the topography, electric current, and friction distributions of the processed areas were discussed. Using the results, we used sequential electrical and mechanical processing to realize the change of electrical resistance due to the formation and removal of the oxide layer.

## Methods

**Figure 1:**
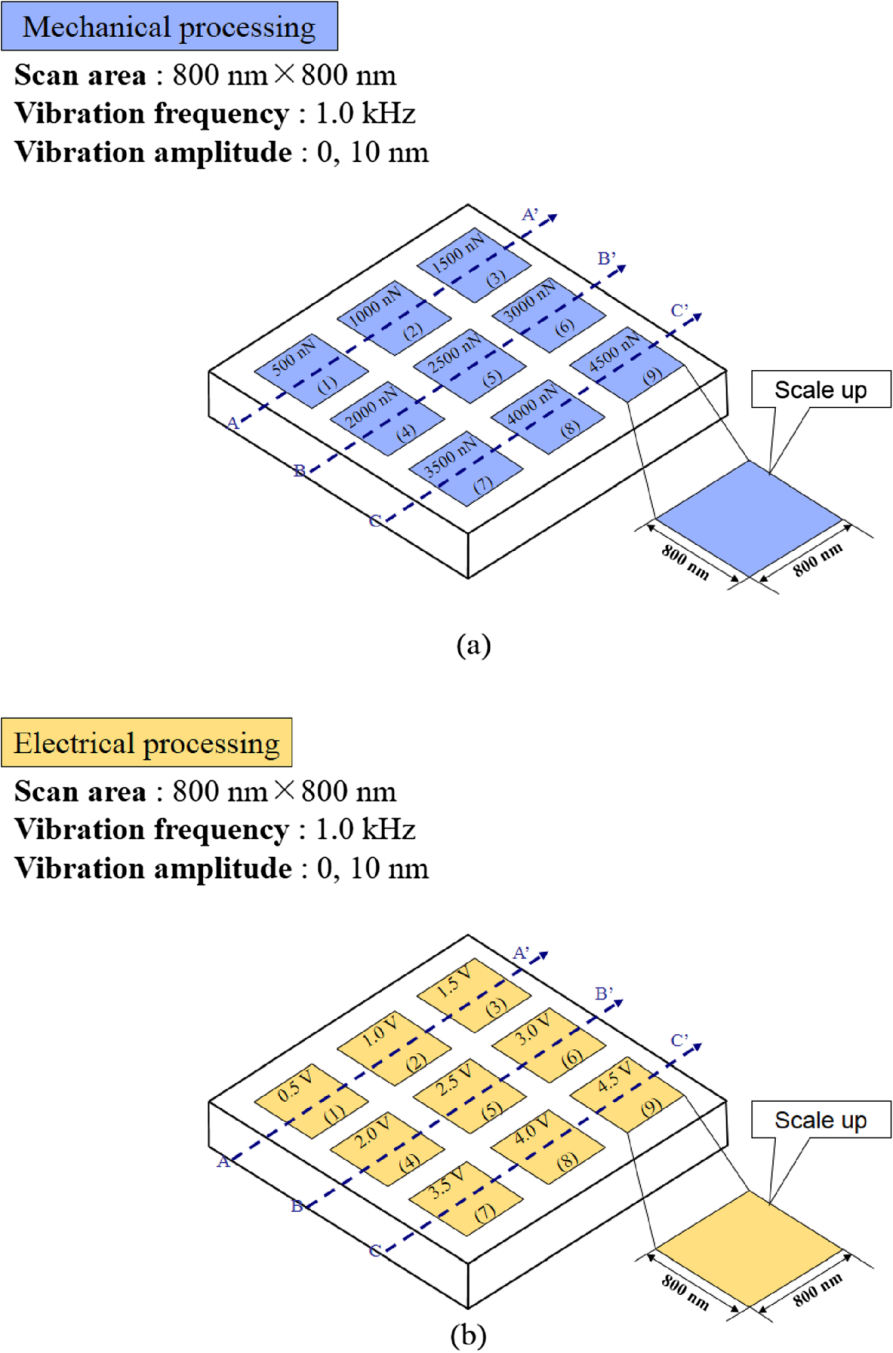
**Conditions for evaluation of mechanical and electrical processing methods. (a)** Mechanical processing at various loads. **(b)** Electrical processing at various voltages.

The specimens used in this study were n-type B-doped Si (100) wafers. The electrical resistance of the wafers was about 4.7 Ω cm (22°C), and their surface was covered with a natural oxide layer. First, nanometer-scale mechanical and electrical processing were performed by tip sliding using an electrically conductive diamond tip with and without vibration at room temperature (approximately 22°C) and 50% to 70% humidity. For the nanometer-scale processing, the conductive diamond tip was used as a processing tool during AFM. Lateral vibration processing was performed using a lateral modulation frictional force microscope (Seiko Instruments Inc., Chiba, Japan) equipped with a cantilever with a diamond tip. Processing was performed by sliding the tip against the sample. During processing, the sample was moved in the scanning direction at a speed of 1.5 mm/s while a vibration of 10 or 50 nm amplitude and 1 kHz frequency was simultaneously applied in the lateral direction [24]. The AFM probe used was a B-doped CVD diamond-coated silicon tip. The radii of the diamond tips were sharp, approximately 45 nm. A schematic of the nanoprocessing method is shown in Figure 1. The processed area was an 800 × 800 nm^2^ square. The system allows topography measurements in contact mode. It is also possible to perform current distribution measurements in contact mode, using a constant bias voltage applied between the sample and the probe. The mechanical and electrical processing were performed under nine sets of conditions. In the case of mechanical processing, scanning was performed by tip sliding with loads of 500, 1,000, 1,500, 2,000, 2,500, 3,000, 3,500, 4,000, and 4,500 nN as shown in Figure 1a. In the case of electrical processing, scanning was performed by tip sliding with voltages of 0.5, 1.0, 1.5, 2.0, 2.5, 3.0, 3.5, 4.0, and 4.5 V as shown in Figure 1b. After processing, the processed surfaces were observed by sliding the same tip at an applied load of about 80 nN and 1 to 3 V within a scanning area of 6 × 6 μm^2^.

**Figure 2:**
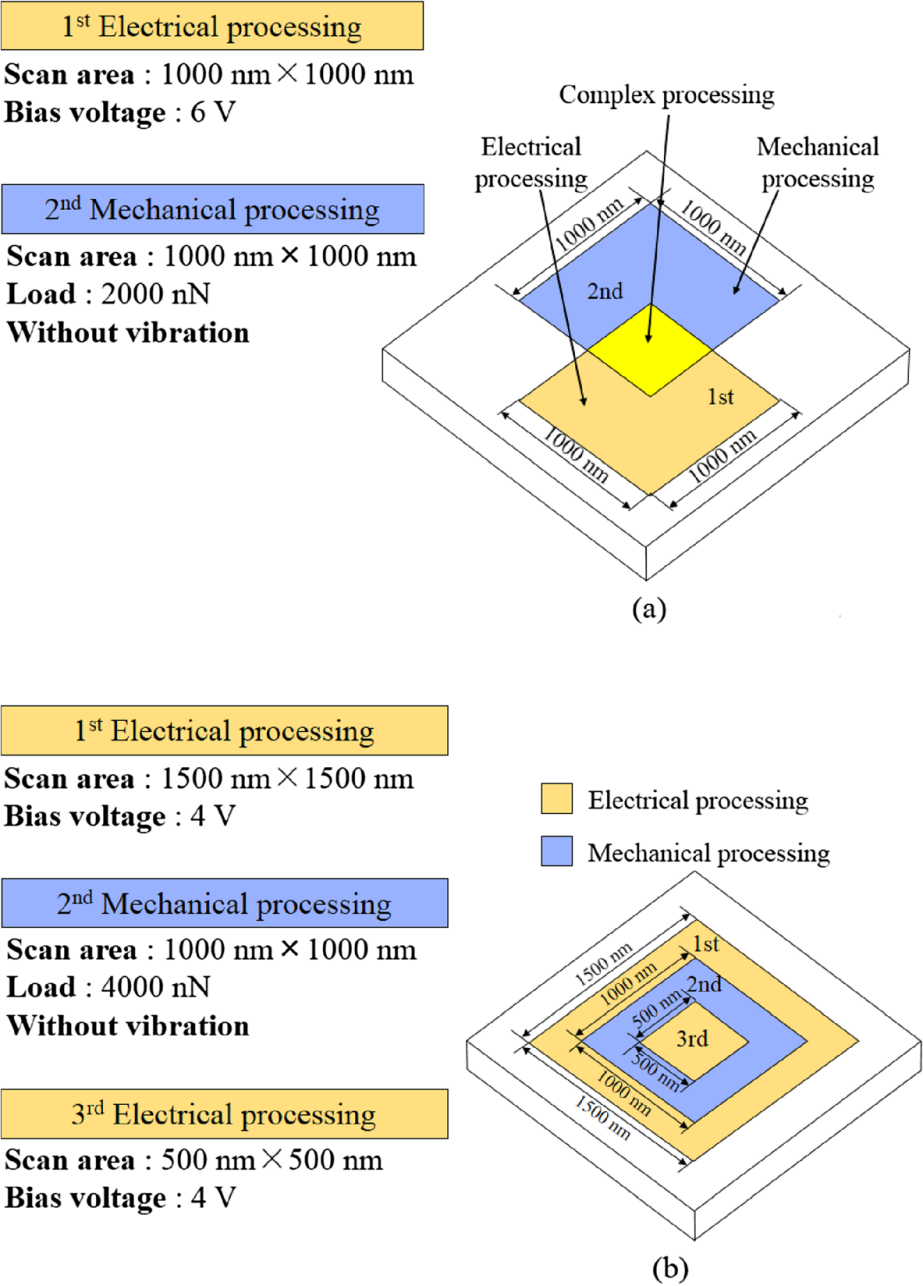
**Sequential processing methods. (a)** Sequential processing A. **(b)** Sequential processing B.

Figure 2 shows the schematics of the sequential electrical and mechanical processing methods. First, the surfaces were subjected to electrical processing as shown in Figure 2a. The 1,000 × 1,000 nm^2^ scan area was processed at 6 V to form a thick uniform oxide layer. The anodically oxidized and unprocessed areas were then processed by mechanical processing at 2,000 nN. Figure 2b shows the three-step sequential processing. First, electrical processing was performed at an applied voltage of 4.0 V. Second, mechanical processing was performed at 4,000 nN load without lateral vibration. Finally, electrical processing was performed again at an applied voltage of 4.0 V. After processing, the profile, electric current, and friction force distributions of the samples were evaluated.

## Results and discussion

### Effect of load on mechanical processing

**Figure 3:**
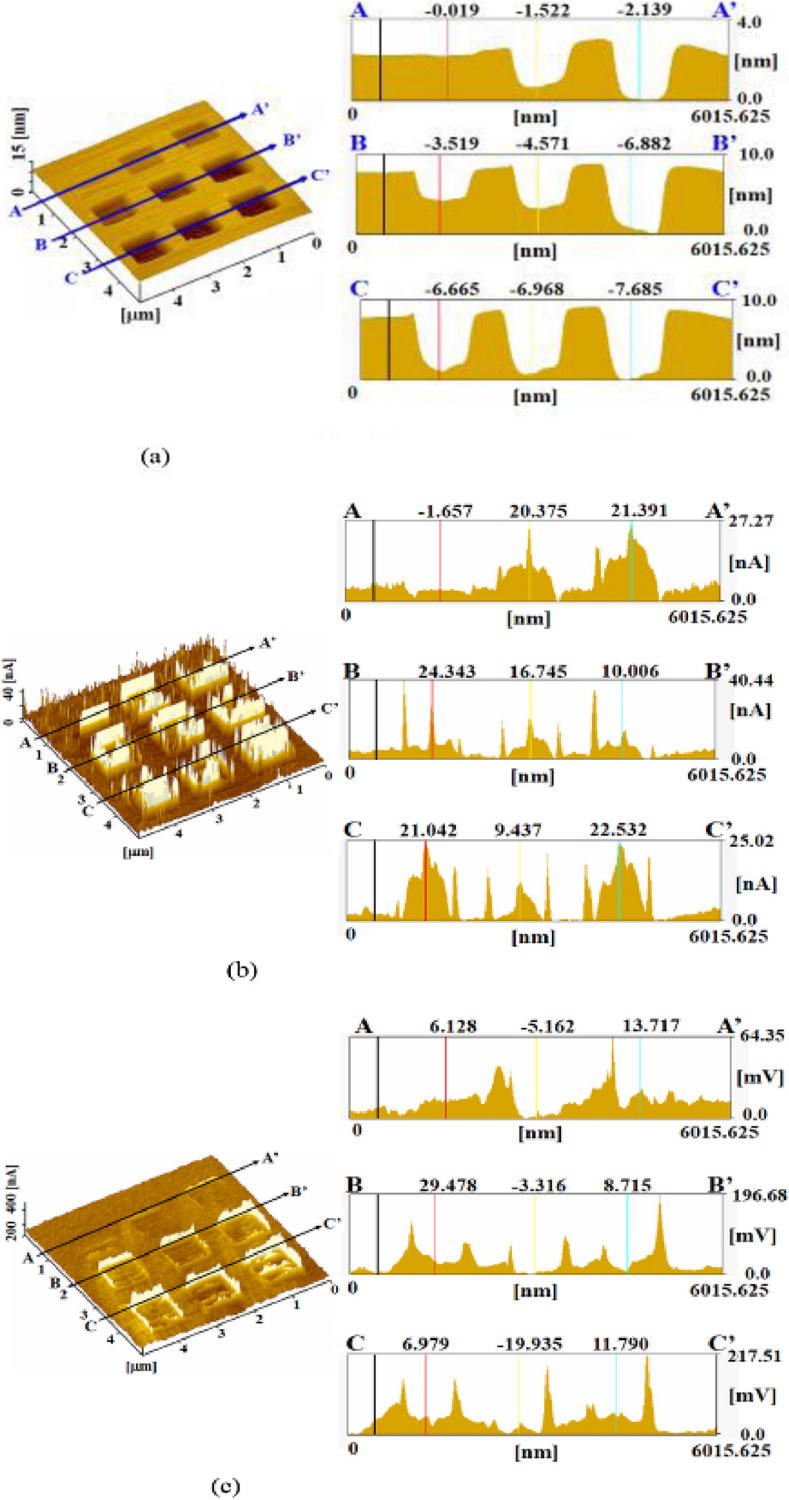
**Dependence of surface properties on applied load during mechanical processing without vibration. (a)** Surface and cross-sectional profiles. **(b)** Current image and profile. **(c)** Friction image and profile.

**Figure 4:**
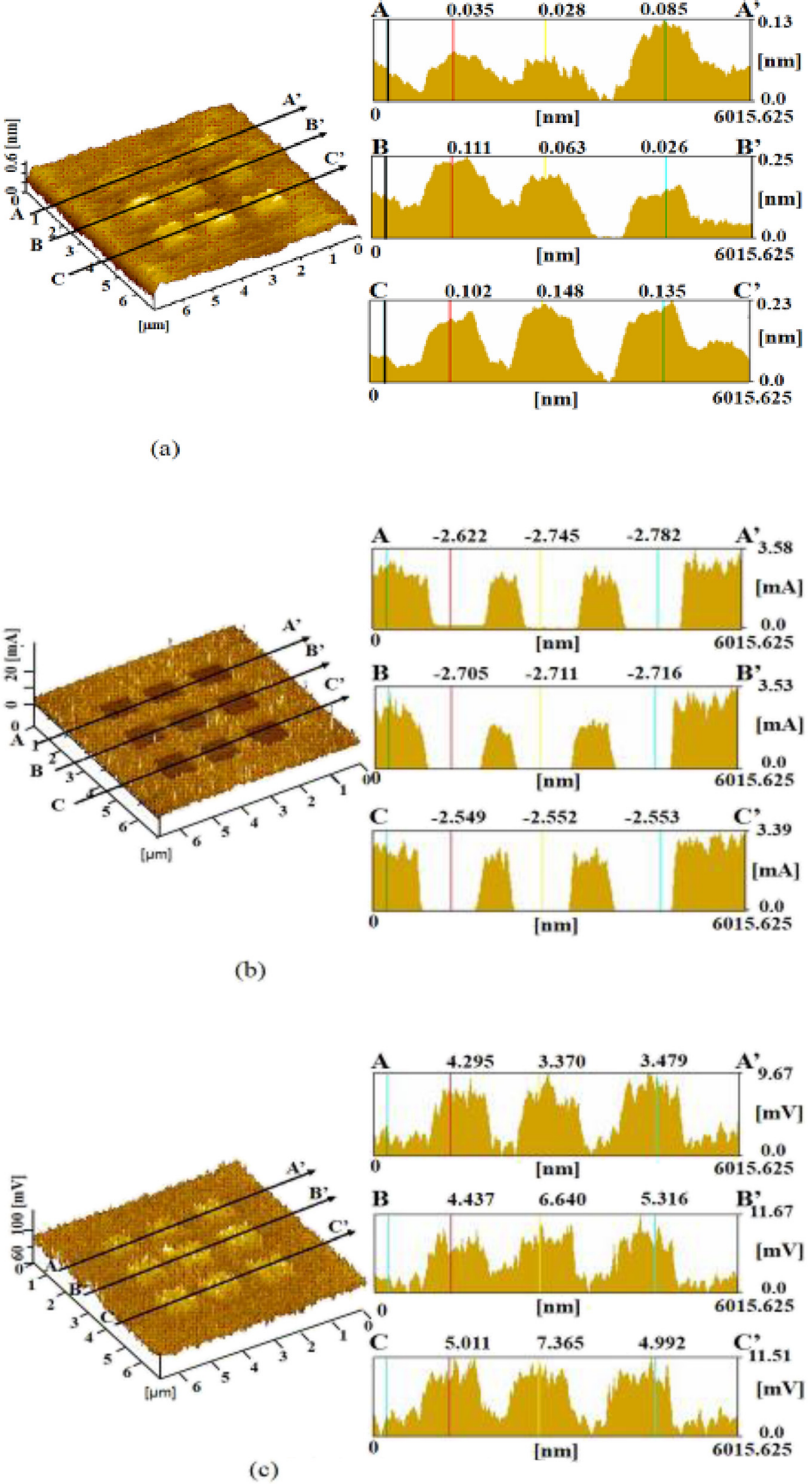
**Dependence of surface properties on applied load during mechanical processing with 10 nm vibration. (a)** Surface and cross-sectional profiles. **(b)** Current image and profile. **(c)** Friction image and profile.

Figure 3a shows the surface profiles of a sample processed under nine different mechanical processing loads without vibration. The mechanically processed profiles are observable as nine square grooves. Groove depth increased with applied load because the depth of the maximum shearing stress increases with applied load [13]. The depth of the groove formed at 4,500 nN load was nearly 7.8 nm. Thus, Si can be removed by processing with a sharp diamond tip because of stress concentration. Figure 3b shows the electric current distribution measured when a voltage of 2.5 V was applied between the tip and the sample. An increase in current compared with the unprocessed areas was observed for the mechanically processed areas, indicating that the natural oxide layers of the silicon were removed by the mechanical processing. The friction distributions were evaluated from the lateral vibration voltage, as shown in Figure 3c. The friction force fluctuated and increased at the edges of the processed grooves. The increase in friction closely corresponds to the observed increase in current and seems to indicate an increase in the strength of the interaction between the diamond tip and the edges of the grooves. Figure 4a shows the surface profiles of areas processed under nine different applied loads with 10 nm amplitude vibration. With vibration, the mechanically processed areas became protruded. The heights of the protuberances were small, at less than 0.15 nm. Protuberance height slightly increased with applied load and reached 0.14 nm at 4,500 nN. In the case of mechanical processing with vibration, the current of the processed areas was decreased, as shown along A-A' in Figure 4b. This current decrease resulted from the mechanochemical oxidization of the surface caused by the applied vibration. Thus, the mechanochemical reaction seems to be advanced by adding vibration. The current of the areas mechanically processed with vibration did not significantly vary with applied load. The friction forces of the processed areas slightly increased as shown in Figure 4c. This was deduced to be caused by the formation of an oxide layer by the mechanochemical reaction with vibration.

### Effect of applied voltage on electrical processing

**Figure 5:**
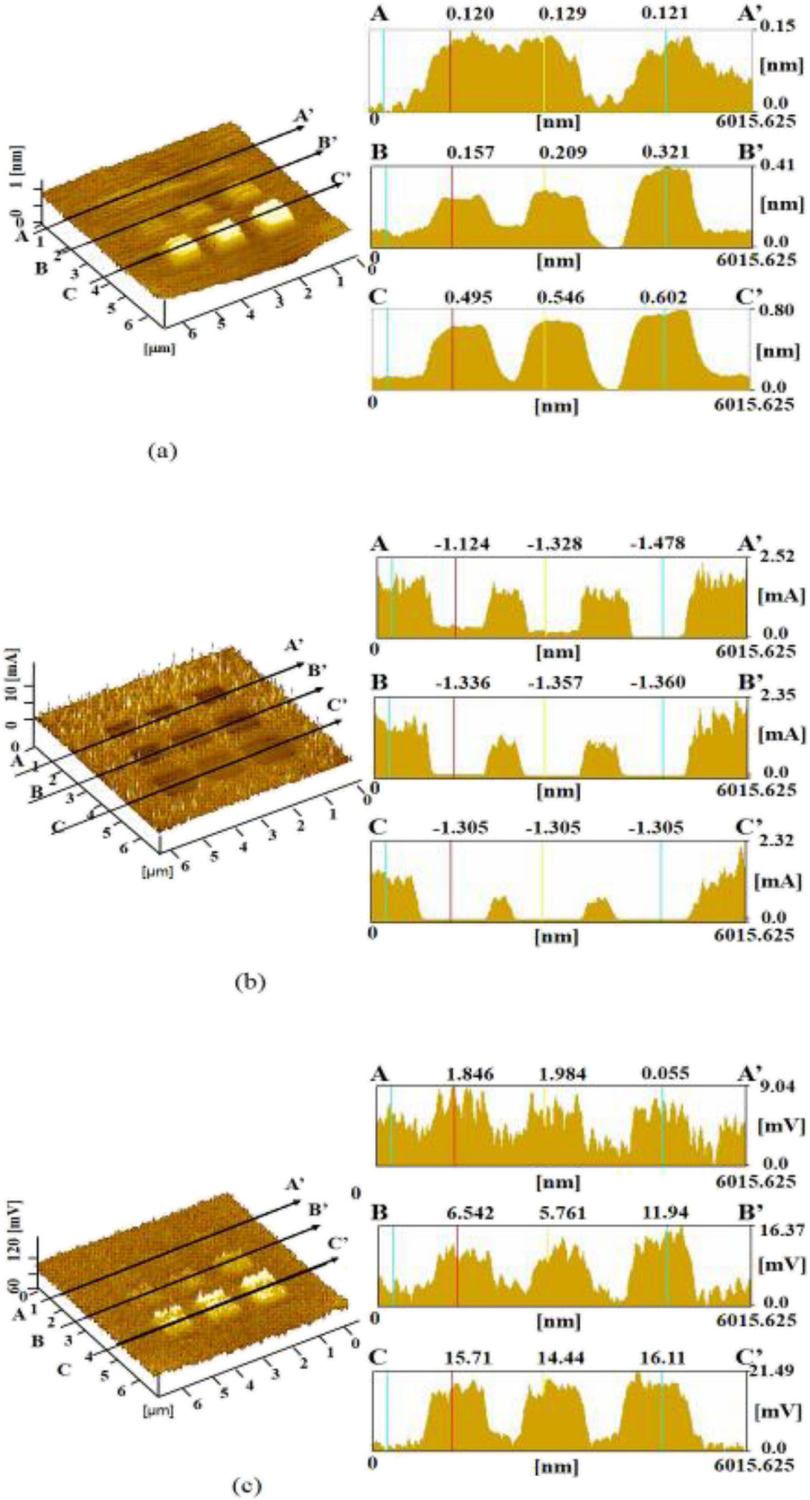
**Dependence of surface properties on applied load during electrical processing with 10 nm vibration. (a)** Surface and cross-sectional profiles. **(b)** Current image and profile. **(c)** Friction image and profile.

**Figure 6:**
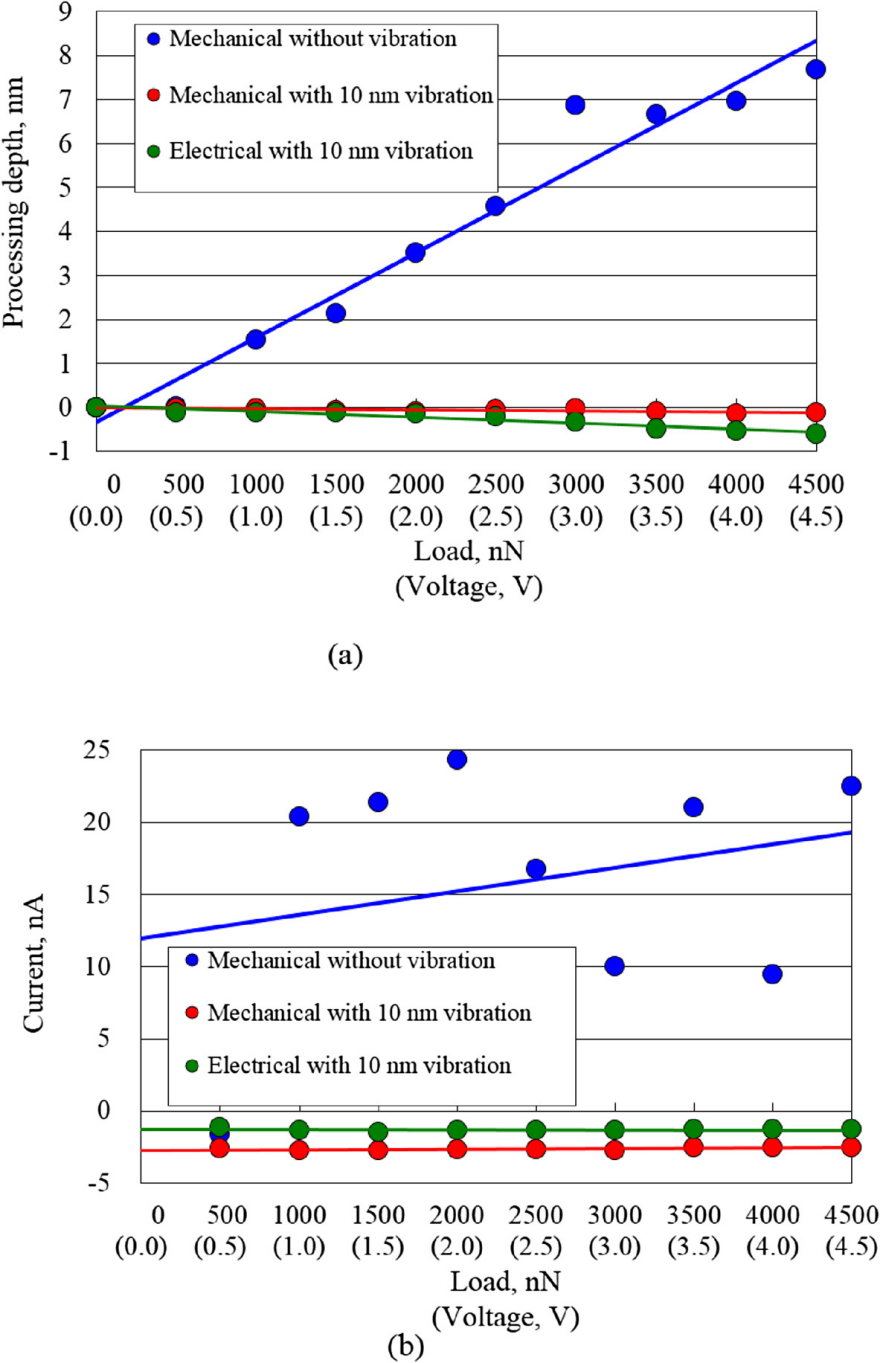
**Dependence of processing depth and surface current on processing load and voltage. (a)** Processing depth. **(b)** Current.

The processed surface and cross-sectional profiles of Si subjected to electrical processing with 10 nm amplitude vibration are shown in Figure 5a. The observed protuberance was formed via the anodic oxidation of Si by oxygen and moisture on the Si surface. The nine squares were processed at applied voltages of 0.5 to 4.5 V. The protuberance height increased with the applied voltage and was 0.12, 0.32, and 0.60 nm at 0.5, 3.0, and 4.5 V, respectively. The protuberance heights of areas processed without and with vibration at 10 and 50 nm amplitudes were about 0.44, 0.60, and 0.71 nm at 4.5 V applied voltage, respectively. The protuberance height increased with vibration amplitude. The heights of the protuberances formed by electrical processing with vibration at a high voltage were higher than those by mechanical processing with vibration.Current distribution images of these electrically processed areas are shown in Figure 5b. The currents measured in the electrically processed areas were decreased compared with that of the unprocessed area as a result of the increase in electric resistance caused by anodic oxidation.The friction force of the electrically processed areas increased clearly with applied voltage, as shown in Figure 5c. The changes in the friction of the areas electrically processed at a high voltage were higher than those observed for the mechanically processed areas. This enhanced increase in friction force was caused by the advance of oxidization with applied voltage.The dependences of the removal depth and protuberance height of the processed areas on load and voltage are shown in Figure 6a. For mechanical processing, remarkably deep removal depths were obtained without vibration up to a maximum of 8 nm. However, when vibration was added, the surface was protuberated by mechanochemical reaction. The maximum height of the protuberances became low, about 0.14 nm. In contrast, the heights of protuberances formed by electrical processing with vibration were higher than those of areas mechanically processed with vibration. The height of the protuberance formed by electrical processing with 10 nm amplitude vibration at 4.5 V was nearly 0.6 nm.

From these results, mechanical processing allows the removal and formation of oxide layers on the surface of Si. The electrical resistance of the surface can be changed by mechanical processing without and with vibration.Figure 6b shows the average difference of currents calculated from the mean current of each processed area minus the current of the unprocessed area. The increase in the current of the areas mechanically processed without vibration was as large as 10 to 25 nA; the current fluctuated and was unstable. In contrast, the mean current change of the electrically processed area was nearly -1.3 nA. The decrease in the current of the area mechanically processed with vibration was -2.5 nA, larger than that of the areas electrically processed with the same vibration amplitude.

### Sequential mechanical processing of electrically processed areas

**Figure 7:**
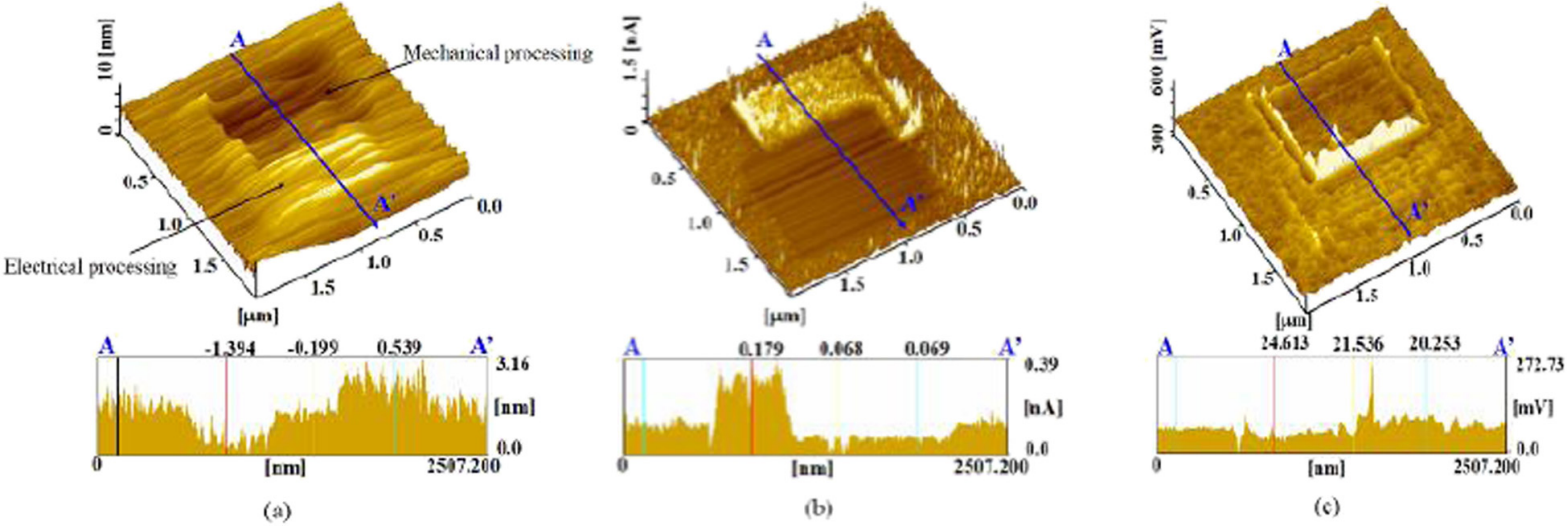
**Surface properties after processing sequence A. (a)** Surface and cross-sectional profiles. **(b)** Current image and profile. **(c)** Friction image and profile.

The surface and sectional profiles of electrically processed and additionally mechanically processed areas without vibration are shown in Figure 7a. A 0.54-nm-high protuberance of the anodic oxide layer was formed by the electrical processing. This protuberance was then removed by the additional mechanical processing without vibration. The depth of the area of the unprocessed surface after mechanical processing was 1.4 nm relative to the initial surface. In contrast, about 0.20 nm of the anodized area was removed relative to the initial surface. The total removal depth of the anodic oxide area was 0.74 nm.The current and friction distribution images of both the anodized and additional mechanically processed areas are shown in Figure 7b,c, respectively. The current of the first electrically processed area decreased because of anodic oxidation. The current obtained after the second mechanical processing was clearly higher than that of the unprocessed surface. The friction force of the mechanically processed area was decreased compared with that of the unprocessed area, as shown in Figure 7c. In addition, the increased friction force of the electrically processed area was decreased by the subsequent mechanical processing. These results indicate that the natural oxide layer of the unprocessed surface was removed by the additional mechanical processing. However, the current of the electrically and then mechanically processed area was lower than that of the unprocessed area. These results suggest that the anodized layer was not completely removed by the additional mechanical processing.

The thickness of the reacted oxide layer can be estimated. Assuming that the electrical processing produced a uniform SiO_2_ layer, the ratio of the thickness of the SiO_2_ and Si layers should be equal to the volume ratio of the SiO_2_ and Si molecules per 1 mol. Therefore, an *x*/0.44-nm-thick SiO_2_ layer is generated from an *x*-nm-thick Si substrate. The total thickness of the SiO_2_ layer is also equal to the sum of the protuberance height and the thickness of the reacted Si. From the average wear depth shown in Figure 7a, the height of the protuberance was nearly 0.54 nm. Thus, the thickness of the SiO_2_ layer should be nearly (0.54 + *x*) nm, where 0.44 (0.54 + *x*) = *x*. The thickness of the reacted Si layer *x* was thereby determined to be 0.42 nm. This 0.42-nm-thick Si layer was then converted into a 0.96-nm-thick SiO_2_ layer by anodic oxidation [25]. In fact, the layer formed as the result of the anodic oxidation contained SiO and damage; therefore, the actual thickness of this layer appeared to be deeper than 0.42 nm. In contrast, with additional mechanical processing, the depth of the electrically processed area became nearly 0.74 nm, thinner than that of the reacted SiO_2_. Thus, it can be concluded that the diamond tip did not remove the total reactive layer. This means that the oxide layer formed by the electrical processing remained even after mechanical processing at 2,000 nN load. In contrast, the removal of the natural oxide layer of unprocessed areas by additional low-load mechanical processing increases the surface current.

**Figure 8:**
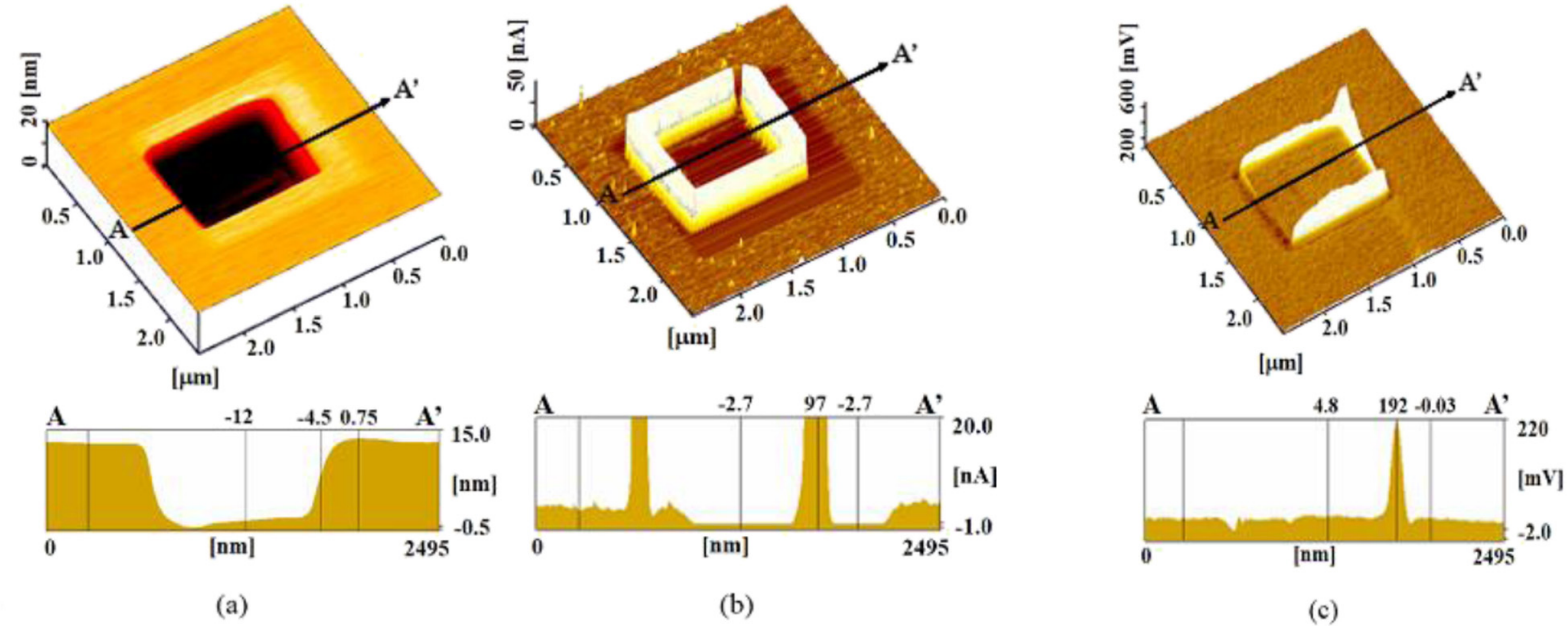
**Surface properties after processing sequence B. (a)** Surface and cross-sectional profiles. **(b)** Current image and profile. **(c)** Friction image and profile.

Figure 8a shows the surface and section profiles obtained after sequential processing B. Electrical processing was first performed at 4.0 V on a 1,500 × 1,500 nm^2^ area. The cross-sectional surface profiles show that the surface of the silicon was oxidized and raised to a height of nearly 0.75 nm by the electrical processing. The center part of the first processing area (a 1,000 × 1,000 nm^2^ square) was then mechanically processed without vibration. Mechanical processing at 4,000 nN load removed the surface to a depth of 12 nm. The electric current of this mechanically processed area was remarkably increased, as shown in Figure 8b. The anodized area was removed by the mechanical processing without vibration, and the electric current increased compared with that of the unprocessed area covered with a natural oxide layer.

It was confirmed that electrically processed areas can be deeply removed by subsequent mechanical processing without vibration. Additional electrical processing caused protuberance at the center of the mechanically processed area; however, the height was as low as <1 nm. It is clear that protuberances formed by electrical processing can be removed by mechanical processing and then reformed by subsequent additional electrical processing. The low height protuberance created inside the mechanically processed area was formed by anodizing.

The currents measured after the electrical processing in the first and third steps were decreased compared with that of the unprocessed surface owing to the anodic oxidation layer formed by the electrical processing. In contrast, the current of the area mechanically processed without vibration was remarkably higher than that of the unprocessed area. Thus, the oxidation layer formed during the first electrical processing was removed by the mechanical processing without vibration, increasing the current. The subsequent electrical processing carried out at the center caused an oxidation layer to reform and decreased the current. Thus, a layer of high electrical resistance can be repeatedly formed and removed by the present processing methods.The friction force increased rapidly at the edge of the groove of the mechanically processed area as shown in Figure 8c, similar to that in Figure 3c. The diamond tip slid at the edge part of the groove, and it is thought that the friction increased owing to the sudden change in shape. The friction of the electrically processed area increased with its detail, and the friction force of the last electrically processed area of the central part was the highest.

## Conclusions

Nanofabrications of silicon by mechanical and electrical processing were performed. The changes of processed profiles, electric current, and friction distribution dependences on processing conditions were evaluated.

1. Nanometer-scale groove and protuberance can be processed by mechanical processing with a sharp and conductive diamond tip without and with lateral vibration, respectively. By mechanical processing without vibration, grooves are formed because of plastic deformation. The natural silicon oxide layer on the surface is removed by the mechanical processing without vibration. Therefore, electric resistance on the surface decreases. In contrast, an oxide layer is formed on the Si surface because of lateral vibration by reacting water or oxygen in the surrounding environment by the mechanochemical reaction. The processed surface protuberates, and their electric resistance increases. Removal and additional oxidization of the surface by mechanical processing can be realized.

2. By electrical processing, the processed surface protuberates and the current decreases by the formation of the oxide layer because of anodization by applying a positive voltage to the silicon. The protuberance height increases with the increase of the applied voltage and the amplitude of the lateral vibration. Decreasing rate in the current of processed surface that was anodized by electrical processing saturated at a certain applied voltage.

3. For sequential processing, the resistivity increases by electrical processing because of anodic oxidation. Then, it is possible to recover the conductivity of the electrically processed area because of removal of the oxide layer by mechanical processing without vibration. The protuberance composed of the oxidation layer processed by the electrical processing can be removed by an appropriate mechanical processing without vibration. The electric resistance of the mechanically processed area can then be increased because of oxidization by additional electrical processing. Repeatable changes of electrical resistance can be performed.

## Competing interests

The authors declare that they have no competing interests.

## Authors' contributions

SM carried out the nanofabrication studies, participated in the nanoprocessing using atomic force microscopy, and drafted the manuscript. SS carried out and evaluated the Si nanoprocessing experiment and helped to draft the manuscript. Both authors read and approved the final manuscript.
